# Emotions and decision-making in boardrooms—a systematic review from behavioral strategy perspective

**DOI:** 10.3389/fpsyg.2024.1473175

**Published:** 2024-11-14

**Authors:** Rosine Hasson Marques, Veronica Violant-Holz, Eduardo Damião da Silva

**Affiliations:** ^1^Faculty of Psychology, Universitat de Barcelona, Barcelona, Spain; ^2^Business School, Pontifícia Universidade Católica do Paraná, Curitiba, Brazil; ^3^Faculty of Education, Universitat de Barcelona, Barcelona, Spain; ^4^International Observatory in Hospital Pedagogy, University of Barcelona, Barcelona, Spain

**Keywords:** strategic decision-making, emotions, board members, emotional influence, organizational behavior, behavioral strategy

## Abstract

This systematic review examines the influence of emotions on strategic decision-making in business context, from the perspective of behavioral strategy. We examined 1,227 articles from two databases (Web of Science and PsycInfo), and after applying the inclusion and exclusion criteria, the final sample resulted in 43 articles. Our systematic review focuses on the role of emotions in strategic decision-making as well as the decision-making process itself. This systematic review explores research using a variety of approaches and a combination of theoretical and empirical perspectives brought by the literature. It aims to address three main questions: how board members’ emotions influence their decision-making; what insights behavioral strategy provides on the emotional aspect of strategic decision-making; and what are the main theories linking emotions to strategic decision-making in the business context. The results demonstrate how emotions can affect the quality of decisions and imply that conflict resolution and emotional intelligence are relevant skills for making strategic decisions. This analysis supports the need for incorporating emotional insights into strategic planning methods by considering agreeable and divergent points of view.

## 1 Introduction

The study of the relationship between emotions and strategic decision-making has become an important area in administration and strategic management in recent years. This recognition represents a shift from the conventional rational-centric perspective to an understanding that accept the part of emotions in decision-making (Brundin et al., 2022). It challenges the traditional view that only rationality influences strategic decisions, highlighting the dynamic interplay between logic and emotion in human behavior.

Researchers like Rodríguez-Cruz and Pinto (2017), Chenli (2022), and Treffers et al. (2020) suggest that emotions and cognition of strategic actors not only coexist but also evolve over time, impacting strategy development and implementation. Studies by Fabio et al. (2023) and Fodor et al. (2016) demonstrate how leaders with high emotional intelligence can influence organizational behavior and strategic outcomes.

Emotions can provide important information that enhances decision-making, rather than merely being irrational reactions that interfere with judgment. Despite technological advancements offering new insights, experts assert that studies have not fully elucidated the emotional effects on individual and collective decision-making among business leaders (Larsen and Stanley, 2021). Cristofaro (2021) highlights a thematic gap, indicating a need for a detailed exploration of how emotions influence corporate strategies and leadership behaviors at senior management levels.

Understanding how emotions impact a board of directors’ behavioral strategy is important because emotions influence how the board interprets information and selects strategic options (Brundin and Nordqvist, 2008). However, little is known about the mechanisms through which emotions affect these processes, highlighting the need for further research. An interdisciplinary approach to behavioral strategy, incorporating insights from organizational psychology, neuroscience, management theories, and corporate governance, is essential for a deeper understanding of emotional dynamics (Butler and Senior, 2007; Powell et al., 2011; Powell, 2017).

Identifying gaps, discrepancies, and inconsistencies in the existing research and current understanding can be facilitated by a comprehensive literature review. Such an examination can pave the way for future studies, guiding scholars and leaders towards more informed and conscientious actions.

Our findings reveal a fragmented knowledge area, highlighting both an opportunity and a necessity for relevant research development. This will contribute to the advancement of knowledge and the consolidation of the field.

Integrating emotional concerns as relevant components rather than distortions that need corrections and incorporating emotion to enhance current theories in strategic management, as noted by Gavetti (2012), Levinthal (2011), and (Powell et al., 2011), justify a systematic review of this subject. This focus may lead to the development of stronger management theories and better corporate governance procedures that recognize the importance of emotions in strategy and decision-making. To guide future research and offer a better understanding and more effective strategies regarding emotions and strategic decision-making in business, we aim to answer three key questions: (1) how do emotions influence board members’ decision-making processes? (2) What insights does behavioral strategy provide on the emotional aspects of strategic decision-making? (3) What are the main theories linking emotions to the strategic decision-making process?

We have conducted a systematic review based on the methodology outlined by Tranfield et al. (2003), following the PRISMA protocol (Moher et al., 2009), to offer a comprehensive synthesis of the literature. This systematic review differs from a scoping review by focusing on in-depth analysis of the influence of emotions on strategic decision-making in boardrooms. As described by Munn et al. (2018), systematic reviews are ideal for addressing specific, clearly formulated research questions and identifying key themes and gaps within a body of literature.

We acknowledge previous systematic literature reviews that have explored the role of emotions in decision-making. For example, Brundin and Nordqvist (2008) conducted a review on boardroom dynamics, which differs significantly from our review in scope, methodology, and focus. While their work explores how emotions shape a broad range of interpersonal interactions and group behaviors within boardrooms, including unconscious emotions and emotional conflicts, our review focuses specifically on the strategic decision-making processes of board members, analyzing how emotions directly impact critical decisions such as decision speed, conflict resolution, and risk assessment from a behavioral strategy perspective.

By concentrating on decision-making processes and integrating elements of psychology, neuroscience, and corporate governance, our review fills a critical gap in the literature. It offers a more targeted analysis of how emotions, particularly emotional regulation and emotional intelligence, influence strategic decisions within boardrooms, complementing broader reviews such as Brundin and Nordqvist (2008), which focus on the general role of emotions in boardroom dynamics.

The future research agenda aims to explore interdisciplinary approaches, such as integrating neuroscience and behavioral psychology, to provide further insights into emotional influences on decision-making. We also suggest longitudinal studies to evaluate how emotional intelligence training impacts the strategic decision-making processes of board members over time.

## 2 Materials and methods

To conduct this systematic literature review, we choose to follow the methodology outlined by Tranfield et al. (2003) and PRISMA. The methodology proposed by Tranfield et al. (2003) for the development of systematic literature reviews expands the applicability of this rigorous and structured method beyond the field of medicine, adapting it to the setting of management research. This approach is divided into three main stages, detailing a step-by-step method that aims to ensure objectivity, transparency, and replicability of the review.

In the initial stage, focused on the planning of the review, the methodology begins with the identification of the need for a systematic review, which is evidenced by the absence of other updated reviews that address the relationship between emotions, strategic decision-making process, and behavioral strategy.

Then we proceed to the preparation of a detailed proposal and the development of a thorough review protocol. This protocol is used for establishing the research extension, as well as creating the inclusion and exclusion criteria, creating strategies for the search and the analysis method.

The second stage, dedicated to doing the review, involves the meticulous identification and selection of relevant research, evaluation of the quality of the selected studies, extraction of data, and finally, the synthesis of the collected data.

The third and final stage focuses on the report and dissemination of the obtained results. According to Tranfield et al. (2003), this stage is important for the conversion of the findings from the review into accessible knowledge, particularly in applied fields such as management. The authors propose the creation of a report in two stages, starting with a detailed descriptive analysis of the field and then proceeding with a thematic analysis that points to the main agreements, disagreements, and emerging issues in the reviewed literature.

Furthermore, we were able to rely on the establishment of a review panel consisting of methodological and theoretical specialists to guide and validate the review process.

**Figure 1:**
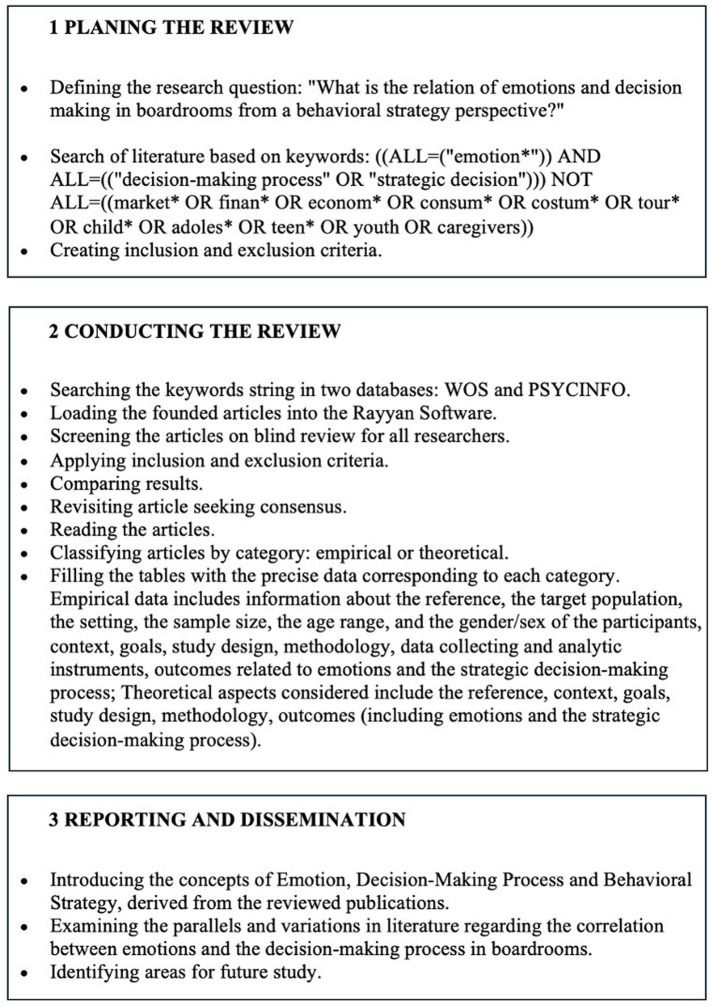
Systematic review method. Based on Tranfield et al. (2003).

By adhering to this detailed methodology, the researchers were able to produce a rigorous and reliable systematic review, contributing to the existing knowledge and providing valuable insights for scholars and practitioners (Figure 1).

## 3 Results

To identify articles that represent the current standing of the literature on the theme of this review, searches were conducted in two databases, namely, Web of Science and PsycInfo. A total of 1,277 articles were located, which were exported and included in the Rayyan platform to automate the screening process.

We utilized Rayyan, a web-based software, to streamline the screening and selection process for relevant studies. Rayyan assists researchers in managing systematic reviews through automated deduplication, inclusion/exclusion criteria filtering, and collaboration features.

Out of these 1,227 articles, 178 were identified as duplicates automatically by the tool. Rayyan offers duplicate resolution for statistically likely duplicates by comparing the title, author, journal, and year. Rayyan was ranked number one for its combination of accuracy and sensitivity for deduplication in an independent third-party study (Rayyan, n.d.). At the end of the automatic duplicate detection process, the software allows for manual review of each potential duplicate, enabling you to confirm whether it is indeed the same article and decide whether to exclude it from the final list or not.

Consequently, 1,049 articles remained for the screening stage, of which one of the authors conducted the initial round of analysis, known as fast screening, checking the entirety of titles, keywords, and abstracts. Following this analysis, to ensure the reliability and validity of the review, the two other authors independently and in blind review, each sampled 10% of the articles.

After the fast screening, there were six articles with disagreement that were resolved by consensus, so 974 articles were excluded for the following reasons: 140 for wrong publication type, 755 for wrong context, 79 for wrong population; the comprehensive search of the remaining 75 articles was undertaken for the second round of analysis. Of these 75 articles, four were not open access and, despite attempts to obtain them from the authors via ResearchGate, were inaccessible, thus analyses were performed on 71 articles.

Upon complete reading of the 71 articles, it was noted that 30 did not meet the inclusion criteria; therefore, two articles were excluded for “Wrong Publication Type,” 13 for “Wrong Population,” and 15 for “Wrong Context.” Consequently, 41 articles remained for analysis. However, the author was aware of two additional articles that met the inclusion criteria through readings and citations, which were then included in the review, totaling 43 articles analyzed in the current literature review.

**Figure 2:**
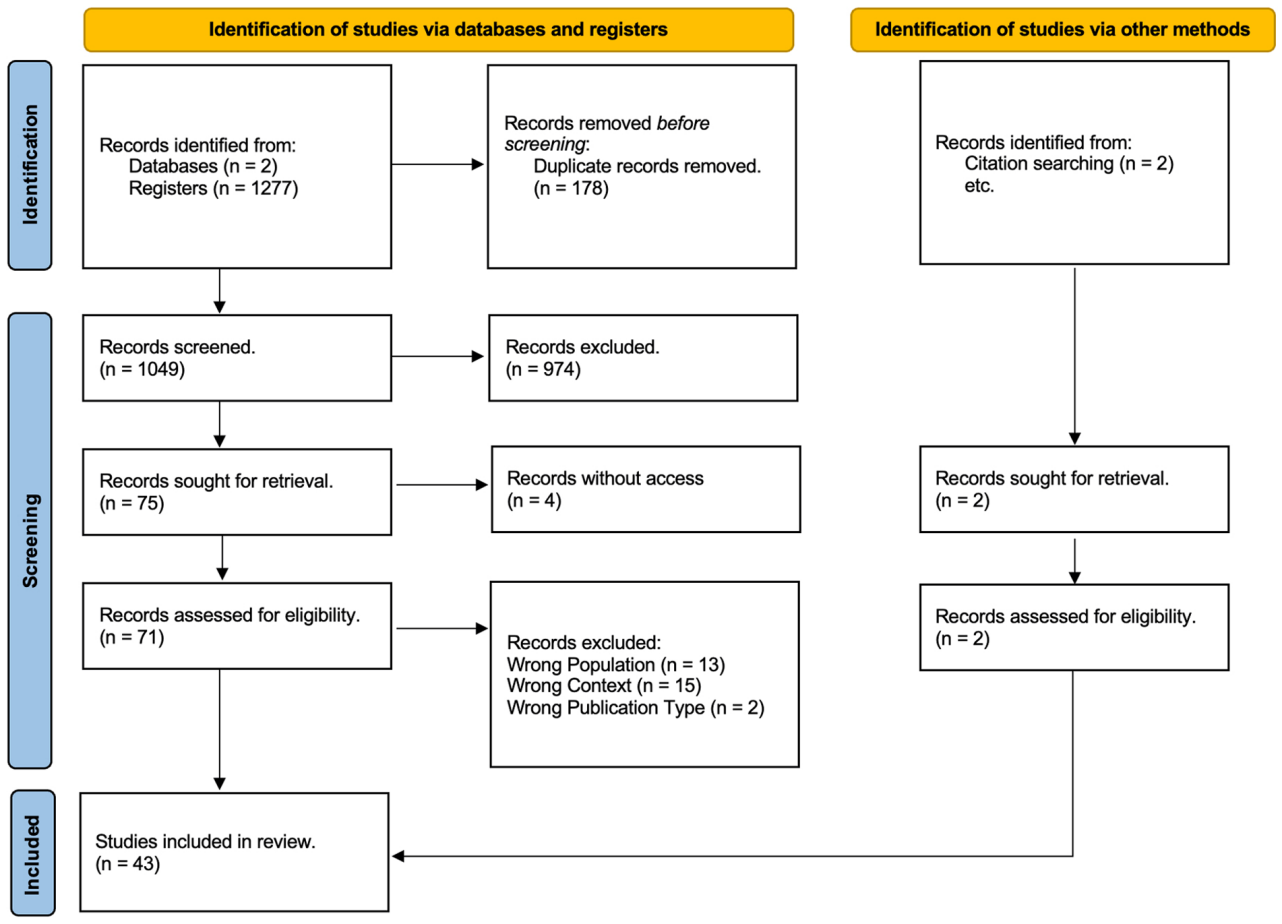
Flow diagram of study selection process.

To illustrate the stages, a diagram was created based on the Preferred Reporting Items for Systematic Reviews and Meta-Analyses (PRISMA) (Moher et al., 2009) (Figure 2).

To ensure methodological integrity and the relevance of our study all the authors used the strict same criteria of inclusion and exclusion, in blind review mode, while doing the first screening that analyzed title, keywords and abstracts of the initial sample. Then a qualitative analysis was conducted to resolve disagreements by consensus in a second round of screening (Page et al., 2021) (Table 1).

**Table 1:** Inclusion and exclusion criteria.

Criteria	Inclusion	Exclusion
Theme	Articles focused on emotions and the strategic decision-making process	Studies not focused on emotions and the strategic decision-making process
Context	Articles with a business context	Articles with contexts other than business
Type of publication	Research articles, systematic reviews, and case studies published in journals	Editorials, commentaries, opinions, book chapters, and conference articles
Abstract	Articles with abstracts	Articles without abstracts
Access	Articles fully available in accessible databases	Articles with restricted access or requiring payment

These inclusion and exclusion criteria were important to maintain the focus on the quality and relevance of the literature selected for analysis.

By excluding articles that were not focused on emotions and strategic decision-making process, we were able to eliminate studies that focused only on one of these two constructs that would not help us to understand the real scenario from the perspective that we conceptualized in this review. The same idea justifies the exclusion of articles that were not on the business context, since our goal is to understand emotions in the decision-making process of board members in cooperatives.

Requiring articles with abstracts enable us to perform an effective preliminary screening of the content and relevance of the studies in relation to the topic of interest. Abstracts provide a condensed overview of the objectives, methods, results, and conclusions of the studies, which is relevant to the initial assessment of an article’s applicability. The stipulation for full open access to articles ensures that our analysis is based on complete data, allowing for an in-depth understanding of the studies and a synthesis of robust evidence.

Furthermore, by excluding articles that require payment or have restricted access, we foster the inclusion of works that are in line with the principles of open access, reflecting the contemporary movement toward more open and publicly available science for both the academic community and the public.

From the final sample, we can establish that all articles included pertain to the strategic decision-making process within a business context.

Although approximately 97% of the initially identified articles were excluded, this can be attributed to the nature of our search criteria, which included articles related to physical and mental health, medical contexts, or emotional relationships and decision-making in social settings—areas outside the business context. The well-defined inclusion and exclusion criteria helped ensure that only studies directly relevant to emotions and strategic decision-making in business were analyzed. This rigorous approach minimizes bias and enhances the reliability of the review methodology (Tranfield et al., 2003).

To show more transparency of the study selection process, we have included Supplementary Table 1, which provides a summary of the included and excluded studies, detailing the reasons for exclusion and inclusion, following the model of D’Oria et al. (2021).

To make it easier to comprehend the findings of these articles, we decided to categorize the outcomes under two main perspectives: Strategic Decision-Making Process and Emotions. The same attempt was made for categorizing articles as empirical articles (Eisenhardt, 1989; Daniels, 1999; Kisfalvi and Pitcher, 2003; Hiller and Hambrick, 2005; Sawers, 2005; Parayitam and Dooley, 2007; Camero et al., 2008; Parayitam et al., 2010; Chen and Ayoko, 2012; Azouzi and Jarboui, 2013; Harbour and Kisfalvi, 2014; Fodor et al., 2016; Meissner and Wulf, 2016; Park et al., 2016; Cruz and Justo, 2017; Rodríguez-Cruz and Pinto, 2017; Palmer et al., 2019; Franke and Foerstl, 2020; Lucena and Popadiuk, 2020; Michel et al., 2020; Netz et al., 2020; Treffers et al., 2020; Ahumada-Tello et al., 2022; Chenli, 2022; Neumann and Wulf, 2022; Vuori and Huy, 2022; Zúñiga Aguilar, 2022; Aschbacher and Kroon, 2023; Balatia et al., 2023; Fabio et al., 2023) and theoretical articles (Ilori and Irefin, 1997; Gaudine and Thorne, 2001; Williams and Miller, 2002; Campbell et al., 2009; Ericson, 2010; Hess and Bacigalupo, 2011; Kaipa, 2014; Putnam et al., 2016; Flores et al., 2018; Brundin et al., 2022; Cristofaro et al., 2022, 2023; Nguyen et al., 2022; Acconito et al., 2023; Yu et al., 2023), so we could analyze them in their specificities. Among the empirical articles, the first evidence that come out is that studies were conducted across different parts of the globe, in total 26 different countries.

From the empirical articles 8 out of 30 used experiments as their design (Fabio et al., 2023; Fodor et al., 2016; Franke and Foerstl, 2020; Meissner et al., 2021; Neumann and Wulf, 2022; Sawers, 2005; Treffers et al., 2020; Zúñiga Aguilar, 2022). Another 6 out of 30 used case study design (Eisenhardt, 1989; Kisfalvi and Pitcher, 2003; Harbour and Kisfalvi, 2014; Park et al., 2016; Lucena and Popadiuk, 2020; Balatia et al., 2023). Some relied on data from experiments or case studies conducted by other authors, and analyzed the data (Hiller and Hambrick, 2005; Rodríguez-Cruz and Pinto, 2017; Palmer et al., 2019; Chenli, 2022; Vuori and Huy, 2022). The diversity of methods can be found in Table 2.

**Table 2:** Main characteristics of the theoretical articles.

Authors	Context	Goals	Study Design	Methodology	Outcomes on Strategic Decision-Making Process	Outcomes Emotions	Journal
Campbell et al. (2009)	Decision-making scenarios and case studies analyzed	To explore and understand why highly qualified and well-intentioned leaders often make flawed decisions.	Case study analysis	Qualitative	Strategic decisions can be significantly influenced by the inherent biases and unconscious processes in human judgment	Emotions play an important role in decision-making through a process the authors refer to as “emotional tagging.” Emotional tags attached to past experiences and memories can significantly influence how decision-makers assess situations and make judgments.	Harvard business review
Ericson (2010)	CEO’s approaches to decision making and emotions	To address the overlooked aspect of incorporating a sense dimension in strategic decision-making research.	Theoretical article	Qualitative	From a strategic decision-making perspective, the main conclusion is that there is a relative neglect in research to include a sense dimension in understanding how managers generate sense when faced with turbulent changes in their environments.	Emotions are much relevant in generating and constituting sense, but this aspect receives little attention in research. Emotions are viewed as providing information that guides and gives meaning, intertwined with the making of sense and anchored in social interaction	Management decision
Gaudine and Thorne (2001)	Ethical decisions in organizations	To develop a model illustrating how emotion influences the components of individuals’ ethical decision-making process, integrating research on arousal and feeling state into a cognitive-developmental perspective.	Theoretical article	Qualitative	–	The model presented in the article clarifies the importance of emotion in the ethical decision process, demonstrating that emotion is intrinsic and not antithetical to a rational ethical decision process, suggesting that attention to one’s emotions may result in better ethical decisions	Journal of business ethics
Ilori and Irefin (1997)	Technology organizations	To discuss the decision-making processes in organizations, particularly focusing on technology decision-making	Theoretical article	Qualitative	The mais outcome from the strategic decision-making process perspective is the importance of combining rational-analytical, intuitive-emotional, and political-behavioral approaches for successful technological decision-making within organizations	From the emotional perspective, the main outcome is the significance of credibility, commitment, and track record in decision-making processes. Proponents need to demonstrate enthusiasm, confidence, and a strategic approach to gain support for technological innovations, especially from senior decision-makers	Technovation
Kaipa (2014)	The stories of the epic Mahabharata	To help leaders develop practical wisdom based on Indian traditions, specifically drawing lessons from the Mahabharata epic to guide decision-making in complex scenarios	Conceptual paper	Qualitative	Leaders need to consider both objective factors like data and logic, as well as subjective factors like emotions, instincts, and intuition to make high-quality decisions	Leaders must develop skills in emotional wisdom, values wisdom, critical thinking, and creative thinking	Journal of management development
Nguyen et al. (2022)	Organizational psychology domain and diversity	To explore the roles of team humor styles and emotional intelligence in the linkage between cognitive diversity and team innovation, utilizing the Conservation of Resources (COR) theory as a theoretical foundation	Theoretical article	Qualitative	Team emotional intelligence (EI) can serve as a valuable resource to resolve conflicts arising from team diversity and ensure a positive team environment for diverse teams	EI allows teams to effectively manage individual and shared emotions, helping to retain members’ self-esteem and images, thus moderating the relationship between cognitive diversity, team humor styles, and team innovation	Organizational psychology review
Williams and Miller (2002)	Executive’s Decision Making	To highlight the importance of tailoring persuasive arguments to the decision-making style of the business leader being targeted, as this significantly increases the chances of proposal success	Theoretical article	Qualitative	Executives tend to have a default style of decision-making that guides them in making tough, high-stakes choices involving complex considerations and serious consequences. This default mode of decision-making is influenced by both reason and emotion, with each decision being a blend of these elements, although the weight given to reason and emotion can vary among individuals	Thinkers, one of the five decision-making styles, are driven by the need to anticipate change and win, often priding themselves on outthinking and outmaneuvering the competition. They have a strong aversion to risk and require comprehensive information, including the presenter’s methodology, to make decisions	Harvard business review

Furthermore, regarding data collection, the studies employed different forms of questionnaires, Likert scale, in addition to semi-structured interviews that had been previously validated. Few articles listed in the table employed *ad hoc* surveys (Daniels, 1999; Park et al., 2016; Rodríguez-Cruz and Pinto, 2017; Aschbacher and Kroon, 2023).

The review identified several key theories in both management and psychology that inform how emotions influence strategic decision-making in boardrooms. Notable theories include emotional regulation (Gross, 1998), the interplay between emotion and cognition (Lerner et al., 2015), and the *Affective Cognitive Theory* proposed by Cristofaro et al. (2022), which posits that decision-making is a product of the intertwined relationship between emotions and cognition. According to Cristofaro, emotions significantly shape cognitive processes, influencing how decision-makers process information and evaluate strategic choices, particularly in complex or ambiguous environments.

From a methodological perspective, most of the studies we reviewed employed qualitative methods, including interviews and case studies. However, several studies also utilized quantitative surveys and Likert scales, which were employed to measure emotional states and their impacts on decision-making processes. Experimental designs, while less frequent, were notable for their use of emotional stimuli to observe changes in decision-making behaviors (Fodor et al., 2016). In terms of data collection, most studies relied on questionnaires or semi-structured interviews, often using validated scales for emotional intelligence or emotional regulation.

Additionally, all articles analyzed in this review are detailed in Tables 2, 3 and Supplementary Table 2, which summarize the main theories and methodologies used. Table 2 outlines the key characteristics of theoretical articles, focusing on decision-making styles, emotional intelligence, and behavioral strategy (e.g., Acconito et al., 2023; Ahumada-Tello et al., 2022). Supplementary Table 2 highlights empirical studies, such as those examining collective emotional intelligence and cognitive biases in decision-making (e.g., Aschbacher and Kroon, 2023). Table 3 summarizes the main characteristics of literature review articles, providing a comprehensive overview of the integration between emotions, decision-making processes, and business strategies.

**Table 3:** Main characteristics of the literature review articles.

Authors, year	N′ Articles and population	Context	Goals	Study design	Methodology	Outcomes on strategic decision-making process	Outcomes on emotions	Journal	Impact factor
Acconito et al. (2023)	20	Decision-making styles, influenced by situational factors and individual differences like emotional and cognitive processes	To propose a multilevel and integrated approach to understanding decision-making styles	Literature review	Qualitative	From the perspective of strategic decision-making, the main outcome is that you have to understand decision-making styles through an integrated approach involving self-report measures, behavioral data, and neuroscientific tools to explore decision-makers’ neuro- and psycho-physiological profiles	Regarding emotions, the main conclusion is that emotional responses are very relevant in decision-making, influencing the evaluation of outcomes and modulating stress during the decision-making process	Neuropsychological trends	(2022) JCI—Psychology—Q4
Brundin et al. (2022)	78	Emotions in strategic management decisions and processes	To expand the previous review focused on strategies by providing an “integrative synthesis” of knowledge on the role of emotions in strategic management overall.	Literature review	Qualitative	The main conclusion from the perspective of strategic decision-making is that emotions are very important role in social interactions among strategic actors, influencing strategic activities like decision-making, planning, change implementation, and failure/turnaround.	From the perspective of emotions, the study highlights the importance of understanding diverse emotion constructs used in strategic management literature, such as discrete emotions, secondary emotions, and predefined constructs borrowed from psychology and organization theory.	Long range planning	(2022) SSCI—Business—Q1 SSCI—Development Studies—Q1
Cristofaro et al. (2022)	23	Affect and cognition interact in managerial decision making	To explore the role of affect and cognition in managerial decision-making processes by analyzing scientific contributions that implement neuroscience techniques or points of view.	Literature review	Qualitative	Affective states can influence cognition, leading to variations in decision-making processes. Managers in a positive affective state under high time constraints generated fewer original and feasible strategic ideas compared to those in a negative affective state, who made better strategic choices	The interplay between affect and cognition is very important in decision-making. Scholars have debated whether emotions influence cognition or vice versa, with some suggesting that both should be studied together as two sides of the same coin.	Frontiers in psychology	(2022) SSCI—Psychology, Multidisciplinary—Q1
Cristofaro et al. (2023)		Exploration of Behavioral Strategy (BS), combining elements of psychology with strategic management.	To synthesize the existing body of research within the field of Behavioral Strategy (BS) and propose a comprehensive and cohesive conceptual framework.	Literature review	Qualitative	Behavioral strategy could be seen as incorporating more reasonable and robust assumptions about human cognition, social passion, and behavior to effectively enhance strategic management within organizations.	Affective states play significant roles in the emotional strategic conduct mechanism, including influencing the content and depth of thought. These states impact the quality of achieved objectives and affect interpersonal responses, shaping the overall strategic direction chosen by leaders.	Academy of management journal	(2022) SSCI—Business—Q1 SSCI—Management—Q1
Flores et al. (2018)	24	Emotional self-leadership and its importance in managing emotions within organizations	To propose a model that highlights the moderating role of emotional self-leadership on the relationship between cognitive conflict and affective conflict within work teams, specifically focusing on its impact on decision quality.	Literature review	Qualitative	Emotional self-leadership plays a crucial role in moderating the relationship between cognitive conflict and affective conflict within work teams, ultimately influencing decision quality positively.	Emotional self-leadership is a comprehensive construct that incorporates elements of emotional intelligence and emotion regulation, providing a holistic approach to addressing emotions and emotionally relevant situations	International journal of conflict management	(2018) SSCI—Communication—Q3
Hess and Bacigalupo (2011)	20	Emotional intelligence in management	To explore how emotional intelligence skills can be practically applied to enhance both individual and group decision-making processes.	Literature review	Qualitative	Emotional intelligence skills, such as self-awareness and self-management, are crucial for decision-makers to determine their role and make effective decisions.	Emotions are very important in decision-making, and decision-makers who understand and manage their own emotions effectively are more successful in the decision-making process	Management decision	(2011) SSCI—Business—Q2 SSCI—Management—Q2
Putnam et al. (2016)	852	Publications that reflected key descriptors of a constitutive approach to contradictions, dialectics, and paradoxes.	To conduct a comprehensive review of literature on contradictions, dialectics, and paradoxes in organizations, focusing on publications that reflect a constitutive approach to these concepts	Literature review	Qualitative	Researchers need to focus more on time in process studies, prioritize emotion over rationality, and explore the interplay between order and disorder in organizations.	Investigations should emphasize the direct link between emotions and workplace stress, burnout, and turnover, highlighting how continual oscillation between poles intensifies negative feelings like anger and frustration	Academy of management journal	(2016) SSCI—Business—Q1 SSCI—Management—Q1
Yu et al. (2023)	26	Family business	To propose a cognitive framework emphasizing the cognitive processes underlying entrepreneurs’ strategic decision-making and their interactions with emotions in Family Business Organizations (FBOs).	Literature review	Qualitative	The main outcomes from the perspective of strategic decision-making include the proposal of a cognitive framework emphasizing cognitive processes in entrepreneurs’ strategic decision-making, particularly in Family Business Organizations (FBOs).	From the perspective of emotions, the article discusses the need to explore the cognitive processes underlying emotional effects and the interactions between emotion and cognition in various social scenarios within FBOs	Long range planning	(2022) SSCI—Business—Q1 SSCI—Development studies—Q1

We also created an image with the software VOSviewer, which is a scientific information network construction and visualization application. The image presents a visual depiction of the terms that appear twice or more in a corpus of 43 articles that were chosen for a systematic literature assessment. This kind of depiction is often used to show conceptual linkages and draw attention to key issues within a particular field of study.

The keywords are dispersed throughout the image’s four primary clusters, as seen by their color and spatial proximity. Emotions, judgment, behavioral strategy, and strategic management are these groupings. Words are sized according to how frequently they occur, with larger words denoting a more significant presence in the dataset under analysis.

Co-occurrences in the articles are reflected in the connecting lines between the keywords, demonstrating the connectedness between ideas both within and across clusters. Interestingly, the term “decision speed” is exceptional; as despite its occurrence in two articles (Eisenhardt, 1989; Treffers et al., 2020), it is exclusively linked to the behavioral strategy cluster, not showing direct connections with other clusters. This implies that “decision speed” may be a new or niche idea, with applicability limited to the behavioral approach context in the collection of literature under analysis.

**Figure 3:**
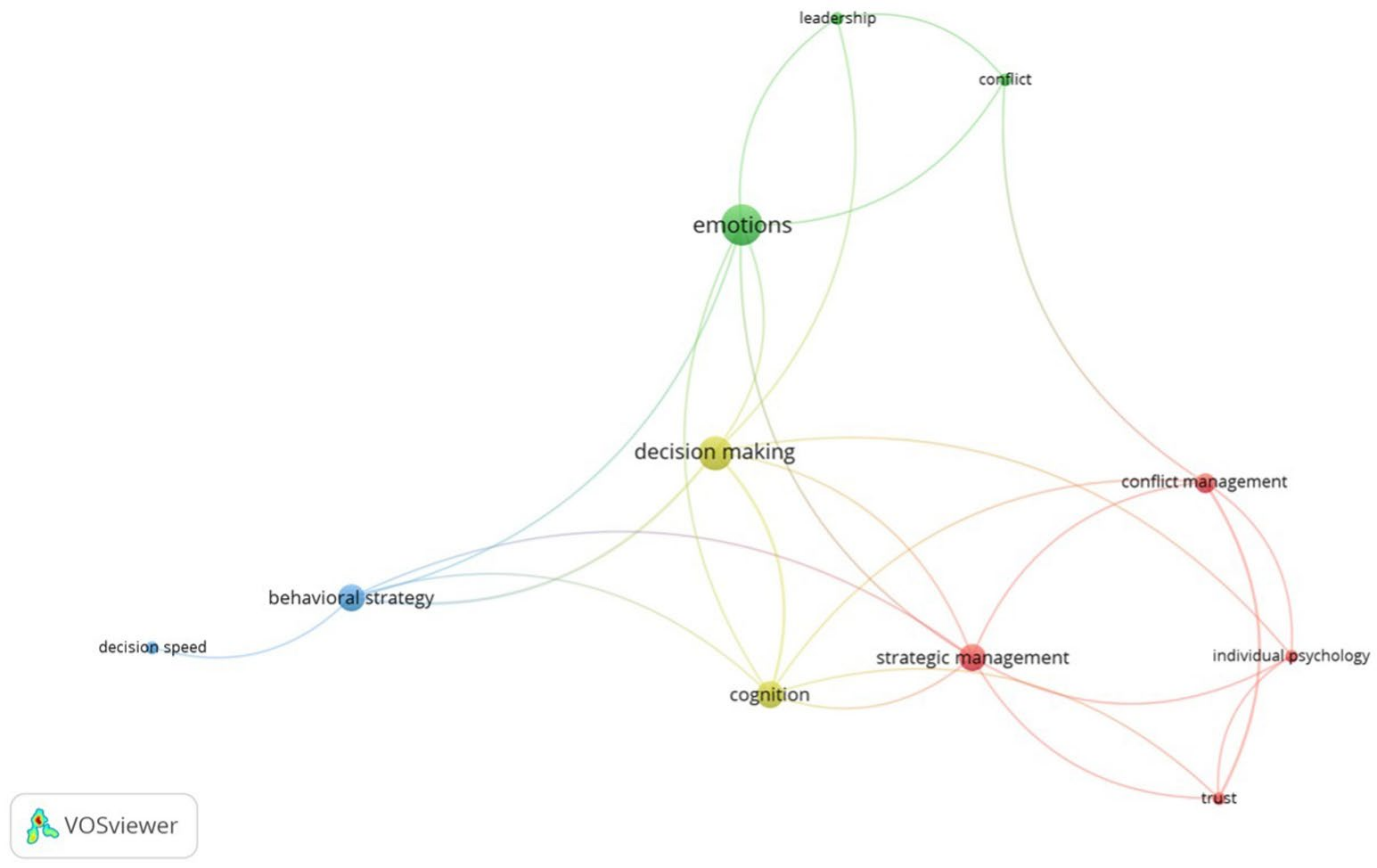
Keywords co-occurrence.

Researchers can identify common areas of interest, potential study gaps, and possibilities for future investigations by using the visual arrangement of the clusters and their relationships, which offers important insights. Large amounts of textual data can be synthesized and interpreted with the help of this kind of keyword co-occurrence analysis, and the graphical representation makes it easier to comprehend the intricate dynamics that define scholarly discourse in the fields of management and organizational behavior (Figure 3).

While examining the complex interaction between emotions and strategic decision-making, we also created another graphic on VOSviewer regarding the publication years. This graphic depiction encapsulates the key findings from the 45 articles that were reviewed. Even though the review covers research from 1989 onwards, what is notable is that the main studies examining how emotions function in the context of strategic decision-making have mostly initiated around 2010.

The information shows how the scholarly community has evolved thematically over time. Starting in 2012, the field of strategic management started to solidify as an important area of scholarly interest, indicating an increase in scholarly activity in this area. There has been a discernible growth in decision-making-related literature since about 2017. This development represents a paradigm improvement in scholarly research by highlighting the decision-making process as an important field of study.

A recent change, which was noticed in 2019, shows that the “Emotions” cluster is growing on relevance. This research’s chronological progression shows that, while being a relatively new phenomena, the consideration of emotions in the context of strategic decision-making has quickly gained traction. Additionally, the convergence of behavioral strategy and emotions was especially emphasized in 2021, which started in a new phase of academic research. These patterns indicate a growing recognition of the importance of emotions in guiding strategic management and decision-making.

**Figure 4:**
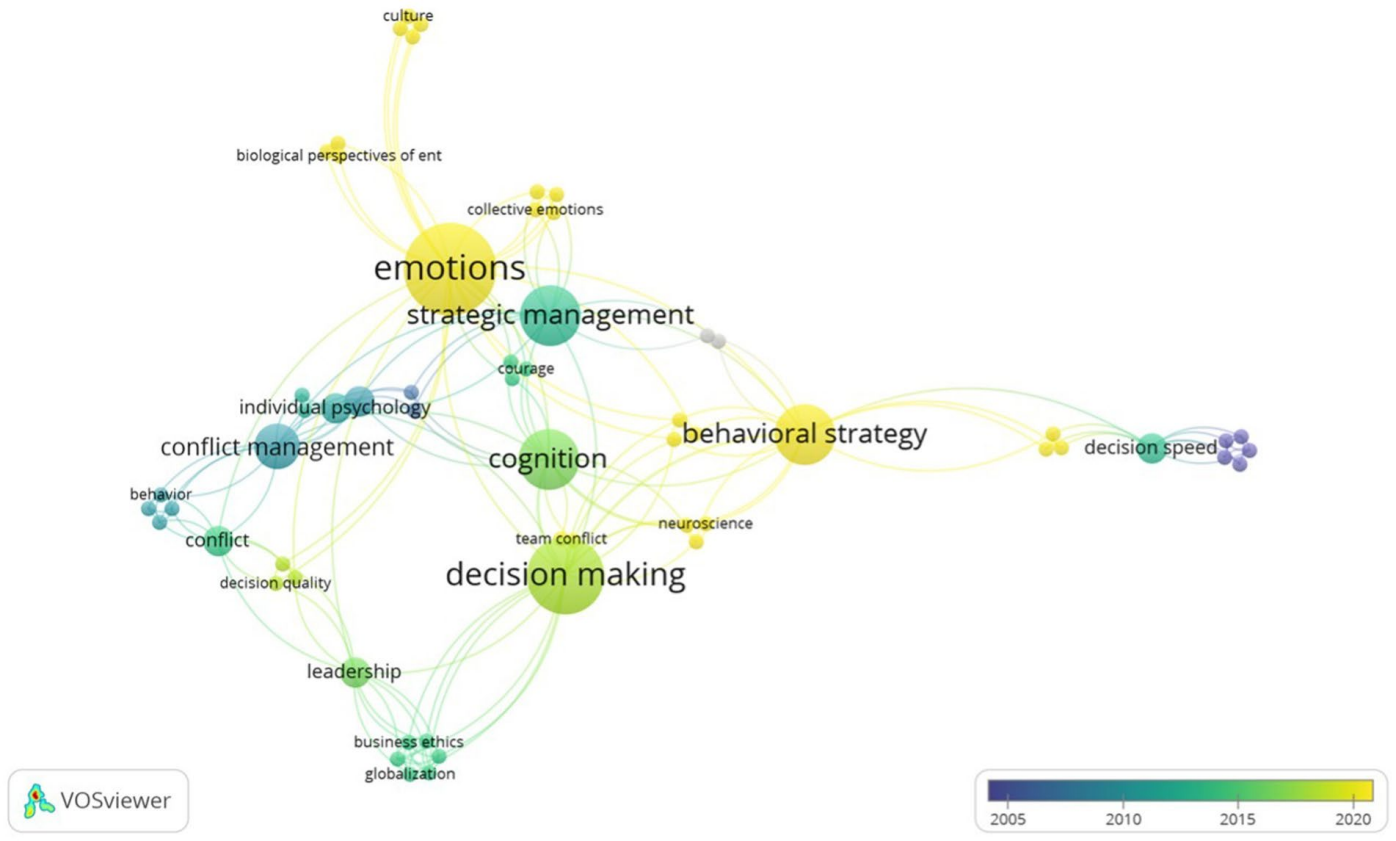
Chronological research evolution.

It is also important to emphasize that from 2020 and on, there is an appearance of “culture” related to “emotions” in the research. This approach shows that culture can influence on the impact of emotions on the interpretation of strategic issues (Neumann and Wulf, 2022) (Figure 4).

The analysis based on the keywords and dates made possible by VOSviewer reveals a promising area of research, with “Emotions” appearing as the newest development in the field of strategic decision-making studies. The gradual integration of affective factors into the conversation marks a change in the direction of scholarly inquiry, creating room for the traditional frameworks to be expanded to include behavioral and psychological aspects within the scope of academic strategic management (Powell et al., 2011). This chronological evolution shows that studies are recently focused on interdisciplinary nature management research, paving the way for more holistic and integrative approaches in future studies. That said, we intend to initiate the search this holistic and integrative approach, by addressing questions that can integrate different areas of knowledge and show what is already known in terms of the influence of emotions and behavioral strategy on the boards’ members decision-making process in the following sections.

### 3.1 How emotions influence the strategic decision-making processes of board members?

The literature review demonstrates that emotions can influence how board members make decisions, impacting both the process and the outcomes. The first question this review seeks to answer is how emotions influence the decision-making process of board members, analyzed systematically to demonstrate how a specific emotion effectively influenced a particular decision.

Ahumada-Tello et al. (2022) found that collective emotional intelligence, shaped by subjective wellbeing and happiness perceptions, dictates organizational decisions. Moreover, Eisenhardt (1989) highlighted how emotions like confidence and anxiety are critical in rapid decision-making contexts. Similarly, Daniels (1999) demonstrated that anxiety and depression influence strategic decisions by skewing focus toward perceived risks and negatively coloring memory.

In contrast, Vuori and Huy (2022) argue that top managers’ decision-making processes were influenced by emotional dynamics. Likewise, Aschbacher and Kroon (2023) highlight in their study on cognitive biases during mergers and acquisitions in Europe how emotions like reluctance and stress directly influence critical decision outcomes.

Additionally, the work of Azouzi and Jarboui (2013) illustrates how emotional intelligence helps mitigate decision biases like loss aversion, enhancing governance effectiveness. Similarly, Balatia et al. (2023) explore the interaction of hope and rage in project retention decisions, emphasizing the complexity of emotional impacts on strategic choices.

The spectrum of emotions influencing decision-making extends to the findings of Camero et al. (2008), where emotional conflicts affected cooperation and competition dynamics. Chen and Ayoko (2012) and Chenli (2022) further explore this dynamic, linking emotional management to trust and strategic decision-making satisfaction, respectively.

On the other hand, Sawers (2005) highlights how choice avoidance in decision-making contexts is driven by negative affect, and Treffers et al. (2020) explore the interaction of time constraints and emotions on strategic decision quality.

Finally, Brundin et al. (2022) suggest that unconscious emotions, defined by the authors as those not always perceived by the individual and potentially related to past emotional experiences, can affect strategic decisions and risk propensity.

Thus, we can see that studies related to emotions show that emotions and their regulation, known as emotional intelligence (Cobb and Mayer, 2000), can influence the decision-making process.

That said, the next proposed step is to understand the emotional aspects of the strategic decision-making process from the perspective of behavioral strategy.

### 3.2 What insights does behavioral strategy provides on the emotional aspect of strategic decision-making process?

In this second question, we seek to analyze the perspective of behavioral strategy on the influence of emotions in board decisions. Behavioral strategy, which integrates elements of psychology with strategic management, offers a lens for understanding the interplay between emotions and decision-making. As articulated by Cristofaro et al. (2023), behavioral strategy embraces a holistic view of human cognition and emotion, positing that affective states influence both the content and depth of strategic thought.

This perspective is supported by empirical findings from studies such as those by Fabio et al. (2023), which demonstrated that induced emotional states affected decision-making styles, particularly in cooperative versus competitive contexts. Similarly, Fodor et al. (2016) found that specific emotions could dictate the choice of heuristics, affecting strategic decision-making among entrepreneurs.

Furthermore, Franke and Foerstl (2020) and Kisfalvi and Pitcher (2003) further support the relationship between emotional states and behavioral strategy, showing how emotional conflicts can create divisions within teams, which can then harm the effectiveness of collective decisions. In the same context, Lucena and Popadiuk (2020) delve into the emotional components of leadership and tacit knowledge in strategic processes.

Moreover, Harbour and Kisfalvi (2014) and Hiller and Hambrick (2005) consider the influence of emotional traits like managerial courage and self-evaluation on strategic decisions. In addition, Chen and Ayoko (2012) demonstrated the mediating effects of emotional arousal on trust within teams, influencing conflict resolution and decision-making quality.

Cruz and Justo (2017) illustrate how family business leaders weigh emotional gains against potential financial risks, demonstrating the deep intertwining of emotional factors with strategic risk assessments. Similarly, Meissner and Wulf (2016) emphasize how anger impacts decision quality, aligning with Michel et al. (2020), who consider emotional dynamics in family-business negotiations.

From a broader psychological perspective, Daniels (1999) discusses how anxiety can skew strategic focus toward risks, impacting overall business strategies. Similarly, the studies by Eisenhardt (1989) and Fabio et al. (2023) emphasize the swift decision-making influenced by confidence and anxiety in high-stakes environments and the behavioral impacts of induced emotional states, respectively.

Palmer et al. (2019) connects psychological traits like dominance and self-efficacy to strategic outcomes in SMEs, while Parayitam et al. (2010) and Parayitam and Dooley (2007) explore trust and conflict dynamics within strategic teams.

Finally, systematic reviews by Acconito et al. (2023), Brundin et al. (2022), Cristofaro et al. (2022, 2023), Flores et al. (2018), Hess and Bacigalupo (2011), Putnam et al. (2016), and Yu et al. (2023) synthesize vast literatures, proposing that emotional-cognition interactions, behavioral strategies, and emotional self-leadership are indispensable for effective decision-making in strategic contexts.

As shown, behavioral strategy highlights the influence of emotions in the strategic decision-making process by integrating psychological elements into strategic management. This sets the stage for the next section, which aims to shed light on the main theories that can enrich the understanding of the interplay between emotions and strategic decision-making.

### 3.3 Which main theories link emotions to strategic decision-making?

Finally, we intend to identify the main theories concerning emotions in the decision-making process of boards. The investigation into the impact of emotions on the decision-making processes of boards of directors reveals a variety of theoretical and empirical perspectives. Our findings show how important emotions are for individual and group decision-making in work environments.

Theoretical frameworks have been proposed to explore emotions in decision-making processes within boards. For example, Campbell et al. (2009), Ericson (2010), and Gaudine and Thorne (2001) provide models and frameworks integrating emotions into the cognitive-developmental perspective of ethical decision-making, suggesting that emotions are not antithetical but intrinsic to making rational ethical decisions. This model challenges traditional notions of rationality by acknowledging the profound impact emotions have on ethical judgments.

Another significant contribution comes from Brundin et al. (2022), who provided an integrative synthesis of knowledge from the perspective of emotions in strategic management. The authors highlight three main theories relating emotions to strategic decision-making: the influence of unconscious emotions, emotional regulation, and collective emotions. The first theory involves the interaction between emotion and cognition, suggesting that emotions are not merely interfering factors in rationality but rather integrated components that influence cognition. The second theory explores emotion regulation, examining how managers can manage their own and their teams’ emotions to facilitate strategic changes and manage resistance to change. According to Thompson (1991, p. 271), emotional regulation refers to the processes responsible for monitoring, evaluating, and modifying emotional reactions throughout life. Additionally, Aldao (2013, p. 155) states that these processes are directly linked to environmental adaptation. The third theory analyzes how shared emotions can influence teams and organizational performance. According to Goldenberg et al. (2020), collective emotions differ from individual emotions as they arise from the dynamics and sense of belonging to a group and occur in relation to a specific event or object.

Moreover, Williams and Miller (2002) discuss decision-making styles influenced by emotional and rational elements, adding depth to the understanding of executive decision-making processes. Furthermore, the review by Acconito et al. (2023) emphasized a multilevel approach to understanding decision-making styles, where emotional responses play an important role in evaluating outcomes and modulating stress during decision-making processes. This underscores the necessity of considering emotional impacts to comprehensively understand and improve decision-making efficacy.

Theories such as Gaudine and Thorne (2001) propose a model where emotions are intrinsic to ethical decision-making, challenging traditional notions that prioritize rationality. This is further supported by the integrative synthesis by Brundin et al. (2022), which discusses the influence of emotions on strategic management activities.

Additionally, the works of Ilori and Irefin (1997) and Kaipa (2014) delve into the integration of intuitive-emotional and political-behavioral approaches in technology and leadership decision-making, highlighting the necessity of emotional wisdom in navigating complex decision landscapes.

The literature also points to nuanced differences in emotional influences across cultures and industries, as seen in studies by Netz et al. (2020) and Neumann and Wulf (2022), emphasizing the importance of contextual and cultural understanding in strategic decision-making processes.

In conclusion, the exploration of the main theories resulting from this systematic review linking emotions to the strategic decision-making process highlights the influence emotions have on both individual and group decision-making within board environments. By integrating emotions into cognitive-developmental perspectives, the reviewed literature underscores the importance of considering emotional factors in strategic management.

As we move forward, the next step is to define the constructs of emotions, strategic decision-making, and behavioral strategy seeking to provide a comprehensive framework to further analyze and understand the intricate relationships between these elements and their implications for effective decision-making within boards.

## 4 Emotions, strategic decision-making process, and behavioral strategy as constructs

As we could see from the results of this systematic review, in management and organizational behavior, emotions, strategic decision-making, and behavioral strategy are closely related ideas. Examining the relationship between each of these fields has helped us better understand how organizations make decisions and how to make those decisions better to achieve better strategic outcomes (Brundin et al., 2022). Therefore, understanding the concept of emotions, the strategic decision-making process, and behavioral strategy is important to bring clarity to the comprehension of the chosen constructs.

### 4.1 Emotions

Emotion can be analyzed from a physiological, behavioral and/or cognitive perspective (Ekman, 1992) is renowned for his work on identifying universal emotions through facial expressions. In his research, he identified six basic emotions: joy, sadness, anger, surprise, disgust, and fear. Ekman argues that these basic emotions are biologically programmed and have implications for decision-making, particularly in high-pressure situations. Recognizing these emotions is important for understanding how automatic emotional responses can influence decision-making.

Basic emotions are defined as innate affective states, characterized by specific facial expressions and unique biological reactions. They are considered universal among humans and, in some cases, observable in other species (Plutchik, 1980). The author also suggested that emotions can vary in intensity and combine to form complex emotional states.

This interpretation of emotions from Ekman (1992) and Plutchik (1980) aligns with the viewpoints of different scholars who have studied emotions’ role in the business context.

Vuori and Huy (2022), regard emotions as a processual experience beginning with the exposure to a stimulus and culminating in a series of internal and external reactions. These reactions encompass not only the affective state and physiological alterations but also extend to the consequential effects on attitudes, behaviors, and cognitive evaluations. Furthermore, these emotional processes are visibly manifested through expressions and other physical signs of emotion, can be pivotal in a strategic business setting.

We also incorporate Kaur (2024) neurological perspective, which emphasizes the importance of emotions in memory formation and decision-making processes within the brain’s structures, specifically the amygdala and hippocampus. This neural foundation of emotions is instrumental in shaping responses to organizational challenges, influencing attitudes toward risk, and affecting the propensity for either innovative ventures or cautious incrementalism.

The construct of emotion used in the article is presented as a complex and integral factor in the strategic decision-making domain. Emotions shapes cognitive processes, behaviors and social interaction within organizations, all of which are important elements in the development and execution of business strategies.

### 4.2 Strategic decision-making process

The strategic decision-making process is a multifaceted concept in business management and organizational strategy.

Bounded rationality is an important concept in behavioral economics and cognitive psychology that challenges the traditional view that individuals always make decisions in a rational and optimal manner. It is based on the idea that humans have cognitive limitations that affect their ability to process information and make fully rational decisions (Simon, 1947, 1955).

On the other hand, strategic decision-making process involves the comprehensive integration of human cognition, emotion, and social behavior to manage and enrich the effectiveness of organizational strategies. According to Huy (2012), strategic decision-making goes beyond the traditional bounds of optimization algorithms that are only focused on judgment and decision-making literature. Instead, it addresses the challenges inherent in strategic management that are characterized by uncertainty and ambiguity. Emotions, particularly, impact both cognition and behavior, especially under conditions fraught with uncertainty and ambiguity (Brundin et al., 2022).

In the innovative context of neurostrategy, as described by Kaur (2024), strategic decision-making evolves further to incorporate the insights from neuroscience. This approach delves into understanding the neural foundation of both conscious and unconscious behaviors, strategic actions, and decisional choices. By focusing on the human brain as the primary level of analysis, neurostrategy emphasizes the influence of emotional and non-conscious cognitive processes in shaping strategic management. The goal is to leverage these insights to optimize organizational competitiveness and performance.

Reflecting on the perspectives of Ashkanasy et al. (2017), the strategic decision-making process also involves the consideration of multiple perspectives to facilitate creative adaptation during times of change.

Healey and Hodgkinson (2017) further elucidate the necessity for emotional and cognitive skills in strategic decision-making, especially within the context of organizational adaptability to changing market conditions. Emotions influence decision-making processes, affecting behavior and reasoning in high-stakes situations. Consequently, executives must adeptly manage both the emotional dynamics and industry knowledge to navigate their organizations through turbulent times successfully.

In conclusion, the strategic decision-making process is a complex construct that requires an integrated approach to management. By incorporating realistic assumptions about human cognition, emotion, and social behavior, and leveraging insights from neurostrategy, this process addresses the inherent challenges of strategic management in dynamic and unpredictable business environment.

### 4.3 Behavioral strategy

Behavioral strategy represents an innovative approach within the field of strategic management, merging insights from cognitive and social psychology with traditional strategic management practices. According to Powell et al. (2011), this interdisciplinary field seeks to ground strategic management in realistic assumptions about human cognition, emotion, and social interaction. By doing so, it aims to enrich both the theoretical underpinnings and the empirical research of strategy theory, as well as amplify the application of these theories in the real-world management of organizations. The essence of behavioral strategy lies in its focus on understanding the human elements that underpin strategic decision-making processes, emphasizing the need for a theoretical foundation that incorporates the complexities of cognitive and social psychology.

Gavetti (2012) further expand on the concept of behavioral strategy by identifying the behavioral roots of superior firm performance. This approach emphasizes the significance of managing cognitive processes to identify and pursue cognitively distant opportunities, which are often less contested and is potentially more rewarding. The author points out the importance of strategic leaders in overcoming the cognitive bounds that prevent firms from seizing these opportunities. This necessitates a broader view of strategic agency, contrasting sharply with more conventional approaches that prioritize local, immediate actions. The focus on mental processes and cognitive distance underscores the potential for behavioral strategy to drive firms toward innovative and less competitive markets.

Levinthal (2011), challenges the traditional divide between economic and behavioral approaches within the strategic field, arguing for a view of rationality as a process rather than a mere outcome. This perspective emphasizes the importance of behavioral mechanisms in the decision-making process, aiming to bridge the gap between rational choice theories and behavioral accounts. Levinthal’s approach calls for the integration of different behavioral approaches and mechanisms into strategic decision-making, with the goal of connecting disparate research strands. This integration is seen as essential for enhancing the field’s ability to tackle practical challenges effectively, suggesting that a more nuanced understanding of behavioral dynamics can lead to more informed and effective strategic decisions.

The collective insights above highlight the core elements of behavioral strategy as an emerging field within strategic management. This approach not only challenges traditional perspectives but also offers a more comprehensive understanding of strategic management through the lens of human behavior. By focusing on cognitive processes, emotional factors, and social interactions, behavioral strategy seeks to provide a more realistic and practical foundation for strategic decision-making. The emphasis on cognitive distance, behavioral mechanisms, and the capacity of strategic leaders in navigating these dimensions signifies a shift toward a more nuanced and human-centric approach to strategic management.

In conclusion, behavioral strategy emerges as a promising framework that integrates cognitive and social psychology into strategic management, aiming to enrich both theoretical and practical aspects of the field. It represents a departure from traditional models by focusing on the human elements that influence strategic decision-making. This approach not only enriches our understanding of strategic management but also opens new avenues for research and practice. As organizations continue to navigate complex and rapidly changing environments, the principles of behavioral strategy offer valuable insights for overcoming cognitive and behavioral barriers, ultimately leading to superior performance and innovation.

## 5 Discussion

As previously mentioned, our objective is to present the articles from three distinct angles: the impact of emotions on the decision-making processes of board members, the correlation between behavioral strategy and the emotions of board members, and the main theories concerning emotions within the decision-making processes of board members. Although we understand that we were able to make arguments upon the three questions, the reviewed literature brought up an important aspect that was common in all three answers, which was the emotional impact on decision-making and how emotional cognition and emotional regulation are fundamental to enhancing the quality and satisfaction derived from strategic decisions (Rodríguez-Cruz and Pinto, 2017; Hesselbarth et al., 2023).

Chenli (2022) discusses how different emotions, including happiness and anger, influence the processing of decision-relevant information. Happiness may reduce the focus on details, while fear can promote a more careful analysis, suggesting that emotions can either cloud or enhance decision quality depending on their nature and management (Bachkirov, 2015). Authors like (Eisenhardt, 1989; Harbour and Kisfalvi, 2014) propose frameworks for mitigating the adverse effects of emotions and emphasize the need for leadership that is emotionally intelligent and capable of navigating complex emotional landscapes.

George and Dane (2016) included mood states, discrete emotions, and affect to this equation demonstrating that positive emotions and affect are not always the best answer when dealing with strategic decision-making, instead, negative affect, feelings, and emotions can sometimes facilitate the decision-making process. The studies suggest that integrating emotional intelligence and cognitive perspectives can enhance decision-making quality.

On the other hand, (Daniels, 1999) shows that negative emotions like anxiety can severely impair decision-making, leading to a more pessimistic view of market conditions and a preference for defensive strategies. This result was supported by (Meissner and Wulf, 2016), who found a negative impact of anger on the quality of strategic decisions.

Regarding emotions, moods, feelings and affect, the Affective Cognitive Theory emphasizes the intertwined nature of emotion and cognition in decision-making and suggest that emotional responses impact persuasion and decision outcomes, highlighting the need for emotional regulation strategies to mitigate negative influences (Kligyte et al., 2013).

While this study primarily focuses on the influence of emotions on strategic decision-making, it is important to acknowledge other important themes that emerge in the literature. For instance, leadership dynamics and its importance in how emotions are managed and expressed within decision-making bodies. Effective leadership often involves emotional intelligence, which is pivotal in navigating group emotions and resolving conflicts in high-stakes scenarios (Goleman, 1995).

According to Goleman (1995) emotional regulation is an important factor of emotional intelligence and translates into the ability that an individual must manage and respond to emotions in a healthy and productive way, preventing, for example, negative emotions from affecting performance and wellbeing. Salovey and Mayer (1990) emphasize the importance of both individual and collective emotional regulation.

The concept of collective emotional intelligence emerges as a theme in our findings. High levels of emotional intelligence within board teams are linked to more adaptive and effective decision-making processes (Fabio et al., 2023). Fodor et al. (2016) and Netz et al. (2020) discuss how emotional competencies within teams enhance the ability to utilize strategic information promptly and adaptively. The literature shows the importance of collective emotional intelligence and its profound impact on organizational performance. High emotional intelligence facilitates better leadership, innovation, and job satisfaction (Ashkanasy et al., 2017).

Emotional intelligence has been discussed as a critical factor in enhancing the quality of strategic decision-making. Studies such as Brundin and Nordqvist (2008) have shown that emotional intelligence improves boardroom dynamics by facilitating conflict resolution and fostering cooperation among board members. Emotionally intelligent leaders can mitigate the negative effects of emotions such as anger and anxiety, which are often detrimental to strategic decision quality (Azouzi and Jarboui, 2013). However, while these findings underline the importance of emotional intelligence in business contexts, recent literature suggests that the influence of emotional intelligence at the strategic decision-making level may still be underexplored.

Coronado-Maldonado and Benítez-Márquez (2023) emphasize that while emotional intelligence is an asset for leadership and team collaboration, its specific role in shaping strategic decision-making processes remains insufficiently understood. Their review demonstrates that although emotional intelligence positively impacts leadership effectiveness and team dynamics, there is still a need to explore how it directly influences the decision-making processes of top management and strategic boards. Specifically, the authors call attention to the importance of further research on how emotional intelligence can be operationalized to enhance decision-making in high-level strategic environments, where the stakes and complexity are often much greater.

As Coronado-Maldonado and Benítez-Márquez (2023) argue, understanding how emotional intelligence informs not only interpersonal dynamics but also cognitive processes related to risk assessment, innovation, and long-term strategic planning is essential for advancing the field. Their findings underscore the need for more empirical research that examines the mechanisms through which emotional intelligence can improve decision outcomes in complex and high-pressure environments.

George (2000) demonstrates that emotional intelligence supports effective leadership by helping people understand and control moods and emotions and improve cognitive functions and decision-making. Gaudine and Thorne (2001) argue that emotional intelligence is essential for effective emotion management, which, in turn, influences organizational decision-making processes. This emotional component is not just supplementary to the cognitive facets of decision-making but is a fundamental aspect that enriches and sometimes steers the decision-making process.

Diverse perspectives exist in the literature regarding how emotions and emotional conflicts impact the process of making strategic decisions. While some research, like that done by Parayitam and Dooley (2007) and Cruz and Justo (2017), indicates that controlling emotions can improve the quality of decisions made, other research, like that done by Azouzi and Jarboui (2013) and Sawers (2005), contends that controlling emotions negatively can skew how people perceive information and result in less-than-ideal decisions.

Additionally, changes in decision-making processes post-crisis have been discussed in the literature, highlighting how emotional stress and uncertainty influence board members’ strategic choices. Studies show that crises often heighten emotional responses, leading to either more conservative or more radical decision-making depending on the emotional climate within the board (Kahneman and Tversky, 1979). Moreover, boardroom behavior, such as group dynamics, power imbalances, and interpersonal relations, are also linked to emotional dynamics and have been shown to impact decision-making quality (Huse, 2007).

These themes are relevant to understand the full scope of how emotions influence decision-making, beyond the specific focus on business contexts. As highlighted by El Beshlawy and Ardroumli (2021), the interplay between emotions and decision-making is complex and multifaceted, involving leadership, organizational behavior, and responses to external shocks.

Our findings show evidence of the important part that emotions take in shaping strategic decision-making processes within boardrooms. As presented, emotional regulation and intelligence are not just supplementary but relevant to enhancing decision quality and organizational outcomes. As our analysis demonstrates, both positive and negative emotions have their importance in decision-making and have different inputs depending on how they are managed. Therefore, fostering emotional intelligence at both individual and collective levels should be a strategic priority for organizations. By integrating these emotional dimensions into strategic frameworks, organizations can achieve more resilient decision-making processes, possibly driving better performance and innovation.

## 6 Limitations

Although this study illuminates how emotions affect strategic decision-making, its relevance might be limited to specific organizational structures or cultural contexts. The diversity of the included publications spans a wide range of settings, making it challenging to generalize these findings to all company cultures or organizational forms.

As a systematic review, this study relies heavily on the quality and rigor of the included papers. The clarity with which the original studies presented their findings and methodologies affects data interpretation. Inconsistencies in study designs and methodological approaches among the evaluated papers may lead to variability in the findings. As well as their conclusions may lose relevance due to changes in the business environment or advancements in management theories, processes and technology over time.

To ensure rigor in the article selection process, we used Rayyan.ai, a semi-automated tool designed to expedite initial screenings in systematic reviews. While praised for its ease of use and time-saving features, Rayyan lacks certain advanced functionalities, requiring manual handling of conflict resolution and data extraction (Kellermeyer et al., 2018), which can introduce variability and bias (Ouzzani et al., 2016). Its machine learning-based suggestions may also lead to overreliance on predictive algorithms, increasing the risk of bias. To mitigate these limitations, we conducted all inclusion and exclusion processes through a blind review, ensuring independent and objective decision-making.

Other limitation that we must acknowledge addresses to the strict inclusion and exclusion criteria, while ensuring rigor, also introduce limitations. The exclusion of approximately 97% of the initial articles was necessary to focus the review on strategic decision-making in business contexts. However, many of the excluded articles dealt with emotions in medical, social, or psychological contexts, which could have provided complementary perspectives on emotional dynamics. By narrowing the scope, the review may have missed insights into how emotional regulation, collective emotions, or decision-making processes function in interdisciplinary settings. While these criteria help reduce bias and ensure focus, they also limit the breadth of the review.

Another limitation regards the databases used—Web of Science and PsycInfo—that are comprehensive but not exhaustive. The choice of these two databases may have restricted the inclusion of relevant studies from other databases or fields.

This study highlights the need for further empirical and experimental research to explore the specific ways emotional states influence strategic decision-making. Developing and validating measurement instruments that accurately capture the impact of emotions on decision-making processes at various organizational levels are also important.

When interpreting the findings of this study and planning future research into behavioral strategy and decision-making, these limitations should be carefully considered.

## 7 Future research suggestions

Future study on the emotional influences on board members’ decision-making processes could concentrate on potential areas, according to the comprehensive review that was provided. To improve our understanding of how emotions affect strategic choices, these fields ought to make use of multidisciplinary approaches that incorporate knowledge from the fields of neuroscience, management science, and psychology.

Future research efforts could focus on conducting experimental and non-experimental studies to methodically investigate the ways in which emotions (such as fear, joy, or rage) impact one’s ability to make decisions. Methods like physiological monitoring and neuroimaging could offer specific information about the physical and neurological reactions related to making decisions under emotional stress. Working together with neuroscientists and psychologists would help these investigations better understand the biological and cognitive processes underlying emotional affect.

Longitudinal research could also evaluate how emotional intelligence training affects the efficacy of decision-making over an extended period. When board members participate in organized emotional intelligence development programs, these studies ought to monitor modifications in the procedures and results of decision-making. Establishing a causal link between better strategic decision outcomes and enhanced emotional intelligence.

While longitudinal studies remain a valuable tool for assessing how emotional regulation evolves over time in boardroom decision-making, future research could also consider integrating physiological and neuroimaging methods. Techniques such as fMRI (functional magnetic resonance imaging) and EEG (electroencephalography) could provide deeper insights into the real-time influence of emotions on decision-making processes (Bechara and Damasio, 2005). These tools can offer a more granular understanding of how emotional states, both conscious and unconscious, affect strategic choices at the neurological level.

Moreover, cross-cultural studies present another fruitful avenue for future investigation. Different cultural contexts shape emotional expression and regulation, which can, in turn, influence decision-making in varied ways (Markus and Kitayama, 1991). By exploring these cultural differences, researchers can provide a more nuanced understanding of how emotions impact decision-making across diverse boardroom settings, allowing for greater generalizability of findings.

A mixed-methods approach, combining qualitative and quantitative data, would further enhance the understanding of these complex dynamics. Surveys, in combination with observational or experimental data, could offer a more comprehensive picture of how board members’ emotional states interact with their decision-making processes (Creswell and Plano Clark, 2018). Additionally, we propose experimental studies that manipulate emotional conditions to assess their causal effects on decision-making outcomes, a methodology that could complement existing observational studies and provide clearer causal inferences.

The significance of emotional conflicts in board dynamics and their effect on the quality of decision-making could be more investigated. The suggested study may employ case study approaches to investigate situations in which emotional conflicts impeded or enabled strategic decision-making. These cases can be analyzed to create frameworks for handling emotional conflicts in high-stakes situations.

In addition to providing fresh perspectives for science, each of these recommended study paths also has real-world applications for raising the moral and ethical standard as well as improving strategic decision-making process at the top organizational leadership levels.

Ultimately, we recommend that future research agendas remain closely aligned with the original research questions, continuously revisiting the central theme of how emotions influence strategic decision-making processes. This integration of longitudinal, cross-cultural, and experimental methods will advance the field and address current gaps in the literature.

## 8 Conclusion

This review has outlined the complex influence of emotions on boardroom strategic decision-making process. We have analyzed a wide range of theoretical and empirical research, which together demonstrate the influence of emotional dynamics on strategic outcomes. The results show that emotions can improve decision-making and are frequently a double-sided, either by improving strategic intelligence through increased empathy and better conflict management or by causing prejudice and impulsivity to bias judgments.

Notably, our review emphasizes how important emotional intelligence is in controlling these affective influences. Emotionally intelligent leaders are better able to handle the complexities of boardroom dynamics and use emotions to create an atmosphere that encourages strategic thinking and action. Additionally, the review gives support to an integrative perspective, arguing that the best processes for making strategic decisions combine emotional intelligence with rational thinking, in line with the latest developments in behavioral strategy and neurostrategy.

In conclusion, understanding emotional dynamics and incorporating them into boardroom processes is not just a way to improve on traditional strategic management; it is also necessary for developing a strong, flexible, and perceptive leadership team. The theoretical field of strategic management is expected to be enhanced by this combination, which also promises to offer useful frameworks that can increase the efficacy of decision-making at the top organizational leadership levels.
